# Biological Profile of Dysmyelopoiesis in Bone Marrow Aspirates at the Joseph Ravoahangy Andrianavalona University Hospital, Madagascar

**DOI:** 10.7759/cureus.110762

**Published:** 2026-06-13

**Authors:** Stephania Niry Manantsoa, Jocia Fenomanana, Tchesterico B Dodoson, Volamahefa Randriambola, Andriamiadana L Rakotovao, Aimée Olivat Rakoto Alson

**Affiliations:** 1 Medical Biology/Hematology, University of Antananarivo, Antananarivo, MDG; 2 Medical Biology, University of Fianarantsoa, Fianarantsoa, MDG; 3 Medical Biology, University of Antananarivo, Antananarivo, MDG; 4 Hematology, University Hospital Joseph Raseta Befelatanana, Antananarivo, MDG; 5 Hematology, University of Antananarivo, Antananarivo, MDG

**Keywords:** bone marrow examination, cytopenia, dysmyelopoiesis, dysplasia, myelodysplastic syndromes

## Abstract

Introduction

Dysmyelopoiesis refers to morphological abnormalities affecting bone marrow cells and may arise in both reactive and malignant conditions. Its interpretation can sometimes be challenging, particularly in settings where access to advanced diagnostic tools is limited. The present study aimed to describe the biological characteristics of dysmyelopoiesis observed in bone marrow aspirates.

Methods

A retrospective descriptive cross-sectional study was conducted at the hematology laboratory of the Joseph Ravoahangy Andrianavalona University Hospital in Antananarivo, Madagascar, over a three-year period from April 2020 to March 2023. Bone marrow smears showing dysplastic features involving at least 10% of cells in one or more hematopoietic lineages were included.

Results

During the study period, 1006 bone marrow examinations were performed, among which 71 cases of dysmyelopoiesis were identified, corresponding to a frequency of 7.06%. Hematological abnormalities were mainly represented by anemia in 55 cases (77.46%), thrombocytopenia in 51 cases (71.83%), and leukopenia in 33 cases (46.48%). Bone marrow cellularity was predominantly normocellular or hypercellular. Dysplasia involved a single lineage in 24 cases (33.80%), whereas multilineage involvement was observed in 31 cases (43.66%). Megakaryocytic abnormalities were mainly characterized by small-sized megakaryocytes in 19 cases (26.76%) and hypolobulated nuclei in 8 cases (11.27%). In the granulocytic lineage, hypogranulation and nuclear hyposegmentation were identified in 26 cases (36.62%) and 18 cases (25.35%), respectively. Erythroid dysplasia was predominantly marked by nuclear budding in 28 cases (39.44%) and laminated cytoplasm in 27 cases (38.03%). Based on the available clinical and laboratory data, dysmyelopoiesis was considered reactive in 18 cases (22.53%) and suggestive of a malignant process in 53 cases (77.46%).

Conclusion

The coexistence of reactive and malignant dysmyelopoietic features highlights the diagnostic complexity of dysmyelopoiesis. Accurate interpretation requires integration of clinical and biological findings and a multidisciplinary approach to guide patient management.

## Introduction

Dysmyelopoiesis encompasses qualitative abnormalities of hematopoiesis affecting the erythroid, granulocytic, and megakaryocytic lineages [1,2]. These abnormalities reflect defective cellular maturation and may include megaloblastoid changes, multinuclearity, hypogranulation, pseudo-Pelger-Huët anomalies, and abnormal megakaryocyte morphology. Such abnormalities may be encountered in a variety of clinical contexts and are not restricted to malignant disorders.

In many situations, dysmyelopoiesis may occur as a reactive and potentially reversible phenomenon, for instance, in the setting of infections, drug exposure, nutritional deficiencies, or toxic insults [3]. Conversely, it represents a key feature of myelodysplastic syndromes (MDS), which are clonal hematopoietic disorders characterized by ineffective hematopoiesis, peripheral cytopenias, and a variable risk of progression to acute leukemia [4].

Although dysmyelopoiesis is widely recognized as an important morphological finding, its overall profile has not been extensively studied outside the context of MDS. Dysmyelopoiesis may also be observed in healthy individuals [1], making the distinction between reactive and malignant dysplastic changes particularly challenging in routine clinical practice, especially in resource-limited settings where complementary investigations are not always available.

In this context, and given the limited data from sub-Saharan Africa, this study was undertaken to describe the biological features and etiological spectrum of dysmyelopoiesis observed in bone marrow aspirates performed in a tertiary university hospital in Antananarivo, Madagascar. By documenting the variety of conditions associated with dysmyelopoiesis, this study aims to improve understanding of this morphological finding and its diagnostic implications in routine practice, particularly in resource-limited settings.

## Materials and methods

This cross-sectional descriptive study was conducted at the hematology department of University Hospital Joseph Ravoahangy Andrianavalona in Antananarivo, Madagascar. The study covered a period of 36 months, from April 2020 to March 2023.

All patients who underwent bone marrow aspiration during this period were considered for inclusion. Cases were retained when dysplastic features were present in at least 10% of evaluable cells in one or more hematopoietic lineages. This cutoff was adopted from the WHO classification criteria for myelodysplastic syndromes [5], in which dysplasia is considered significant when present in at least 10% of cells within a given lineage. Although our study was not designed to diagnose MDS, this threshold was used as a standardized morphological criterion to identify clinically relevant dysplastic changes regardless of their underlying etiology.

Morphological assessment was performed using standard criteria. For each case, at least 200 cells were evaluated for the erythroid and granulocytic lineages, while a minimum of 30 megakaryocytes were assessed.

All bone marrow smears included in the study were reviewed independently by two experienced hematologists. When differences in interpretation occurred, the slides were jointly reviewed, and a consensus diagnosis was established. Formal inter-observer agreement was not assessed.

The etiological classification of dysmyelopoiesis was based on the integration of bone marrow morphological findings, clinical presentation, and available laboratory data. Reactive dysmyelopoiesis was assigned according to the clinicobiological context and the absence of evidence supporting a clonal hematological disorder, whereas malignant dysmyelopoiesis corresponded to cases associated with hematological malignancies diagnosed according to the available clinical, biological, and morphological criteria.

Peripheral blood counts were performed using an automated analyzer with a five-part differential, Mindray BC 5380® (Mindray Bio-Medical Electronics Co., Ltd., Shenzhen), on blood samples collected in EDTA tubes. Peripheral blood smears were prepared according to routine laboratory procedures and stained using the May-Grünwald-Giemsa stain. Reticulocyte counts were performed in patients with anemia using supravital staining with brilliant cresyl blue. Bone marrow aspirates were obtained from sternal or iliac sites, and smears were prepared immediately after aspiration before staining to preserve optimal cellular morphology for dysplastic evaluation.

It should be noted that additional investigations, including cytogenetic analysis, molecular studies, flow cytometry, and Perls staining, were not available in our setting. The retrospective design and the lack of systematic iron, vitamin, cytogenetic, and molecular investigations may have led to some degree of etiological misclassification, particularly among cases classified as reactive dysmyelopoiesis.

Given the retrospective nature of this study, which was based exclusively on the analysis of pre-existing anonymized medical data without any intervention or direct patient interaction, ethical committee approval was not required in accordance with institutional regulations and current international guidelines.

## Results

Over the study period, a total of 1006 bone marrow examinations were performed. Among these, 71 cases met the inclusion criteria, corresponding to a frequency of dysmyelopoiesis of 7.06%. The age of patients presenting with dysmyelopoietic features ranged from 3 to 85 years, with a mean age of 50.15 ± 21.04 years (median age, 58 years). Patients older than 60 years represented the predominant age group (49.29%, 35/71), followed by those aged 45-60 years (19.72%, 14/71). Patients aged 15-30 years accounted for 11.27% (8/71) of the cases, whereas patients younger than 15 years and those aged 30-45 years each represented 9.86% (7/71) of the study population. Hematological abnormalities were common and are summarized in Tables 1-3.

**Table 1:** Quantitative Characteristics of Complete Blood Count (71 cases)

Parameters	Findings	Number of cases (N=71)	Proportion (%)
Hemoglobin (reference range: females, 11.5-16.5 g/dL; males, 12-18 g/dL)	Anemia	55	77.46
	Normal	16	22.54
	Polycythemia	0	0.00
Mean corpuscular volume (MCV; reference range: 80-95 fL)	Microcytosis	11	15.49
	Normocytosis	43	60.56
	Macrocytosis	17	23.94
Mean corpuscular hemoglobin concentration (MCHC; reference range: 32-36 g/dL)	Hypochromia	11	15.49
	Normochromia	60	84.51
White blood cell count (reference range: 4.0-10.0 x 10^9^/L)	Leukopenia	33	46.48
	Normal	19	26.76
	Leukocytosis	19	26.76
Platelet count (reference range: 150-450 x 10^9^/L)	Thrombocytopenia	51	71.83
	Normal	16	22.54
	Thrombocytosis	4	5.63

**Table 2:** Hematological Parameter Values (71 cases)

Parameters	Minimum values	Maximum values	Mean values	Median values
Hemoglobin level (g/dL)	3.9	18	9.5	9.4
Mean corpuscular volume (MCV) (fL)	58	124	88.11	86
White blood cell count (×10⁹/L)	0.2	358.0	24.9	4.3
Platelet count (×10⁹/L)	4	729	123.35	63

**Table 3:** Qualitative Features of Peripheral Blood Smears (71 cases)

Qualitative abnormalities	Present	Absent or not reported
Red blood cells		
Anisocytosis	15	56
Poikilocytosis	19	52
Basophilic stippling	7	64
Leukocytes		
Hypo- or hypergranulation	31	40
Nuclear segmentation abnormalities	48	23
Platelets		
Giant platelets	14	57
Circulating micromegakaryocytes	3	68

**Figure 1:**
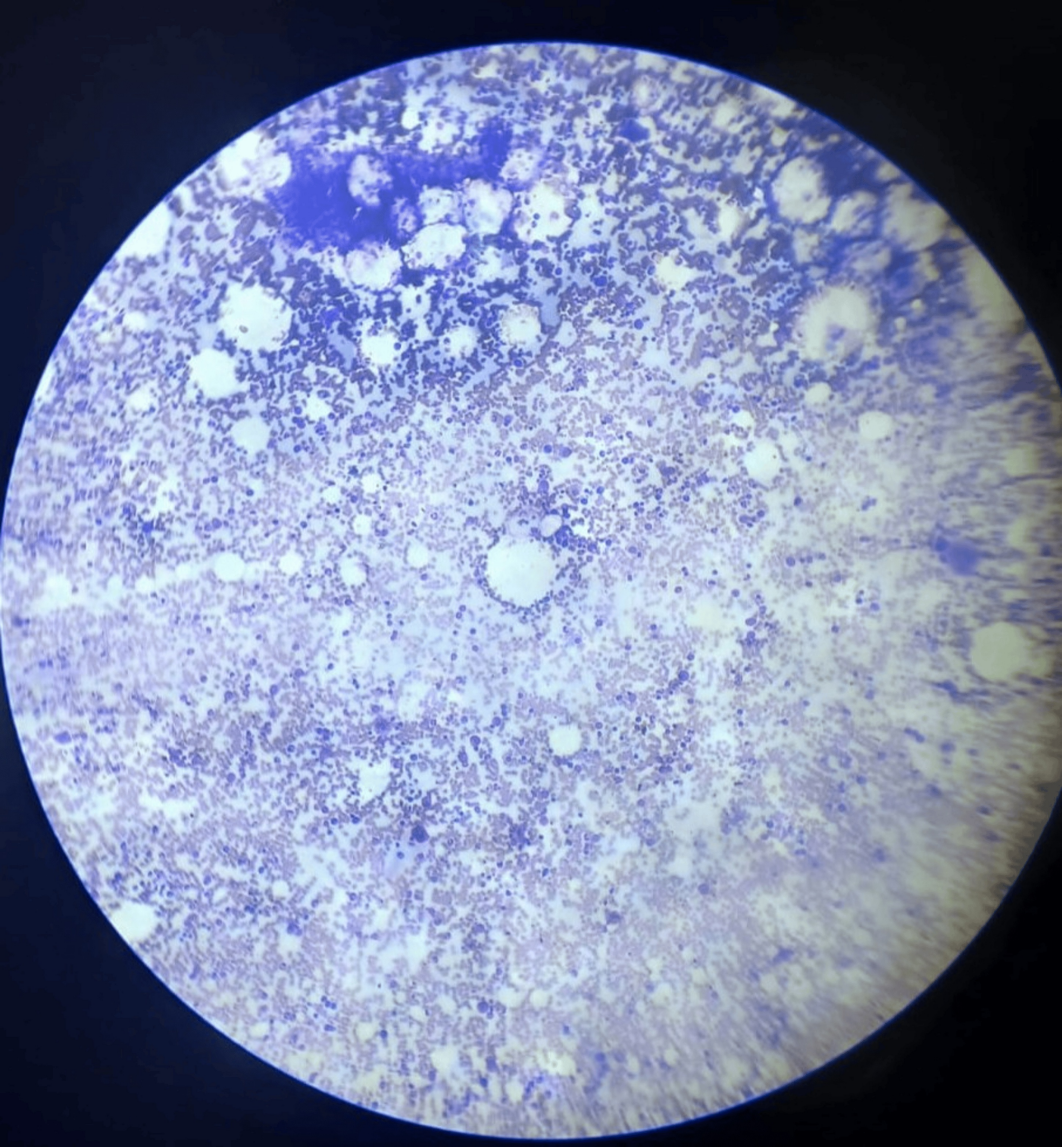
Hypercellular bone marrow Bone marrow smear under light microscopy, 10× objective, using May-Grünwald-Giemsa staining

Anemia was the most frequently observed finding (77.46%), followed by thrombocytopenia (71.83%) and leukopenia (46.48%). In most cases, anemia was either normocytic or macrocytic. Among the 55 patients with anemia in whom reticulocyte counts were assessed, the majority presented with non-regenerative anemia (91.84%). Regarding bone marrow cellularity, normocellular and hypercellular patterns (Figure 1) were each observed in 33.8% of cases, whereas hypocellularity accounted for 4.23%.

**Figure 2:**
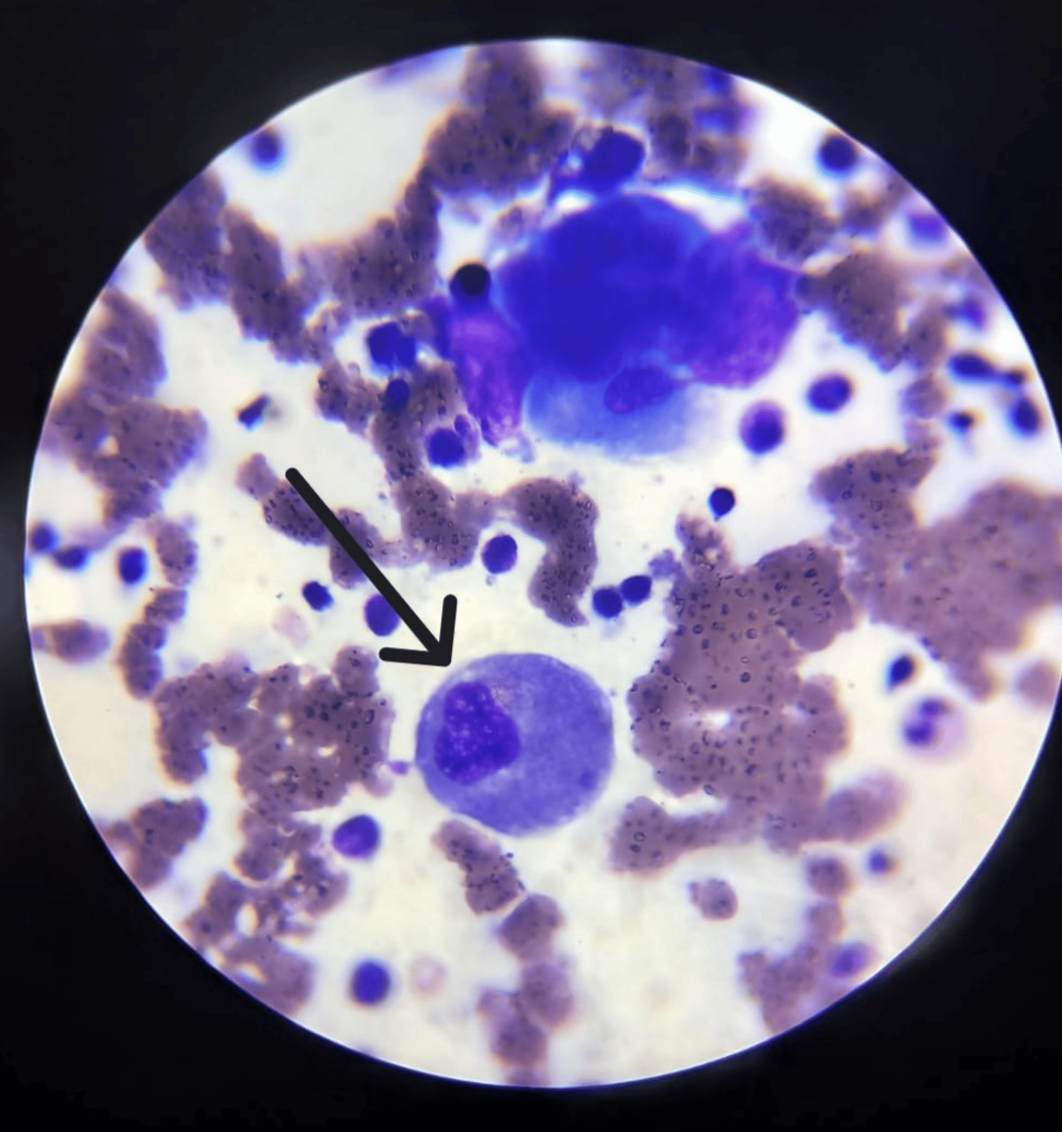
Dysmegakaryopoiesis - hypolobulated nuclei, small-sized megakaryocytes Bone marrow smear under light microscopy, 100× oil immersion objective, using May-Grünwald-Giemsa staining The arrow indicates a small-sized megakaryocyte with a hypolobulated nucleus.

Among the 55 bone marrow aspirates with a sufficient number of megakaryocytes for dysplasia assessment, megakaryocytic dysplasia was predominantly characterized by small-sized forms (26.76%) and hypolobated nuclei (11.27%). The coexistence of several types of abnormalities was observed in nine patients (12.68%) (Figure 2).

**Figure 3:**
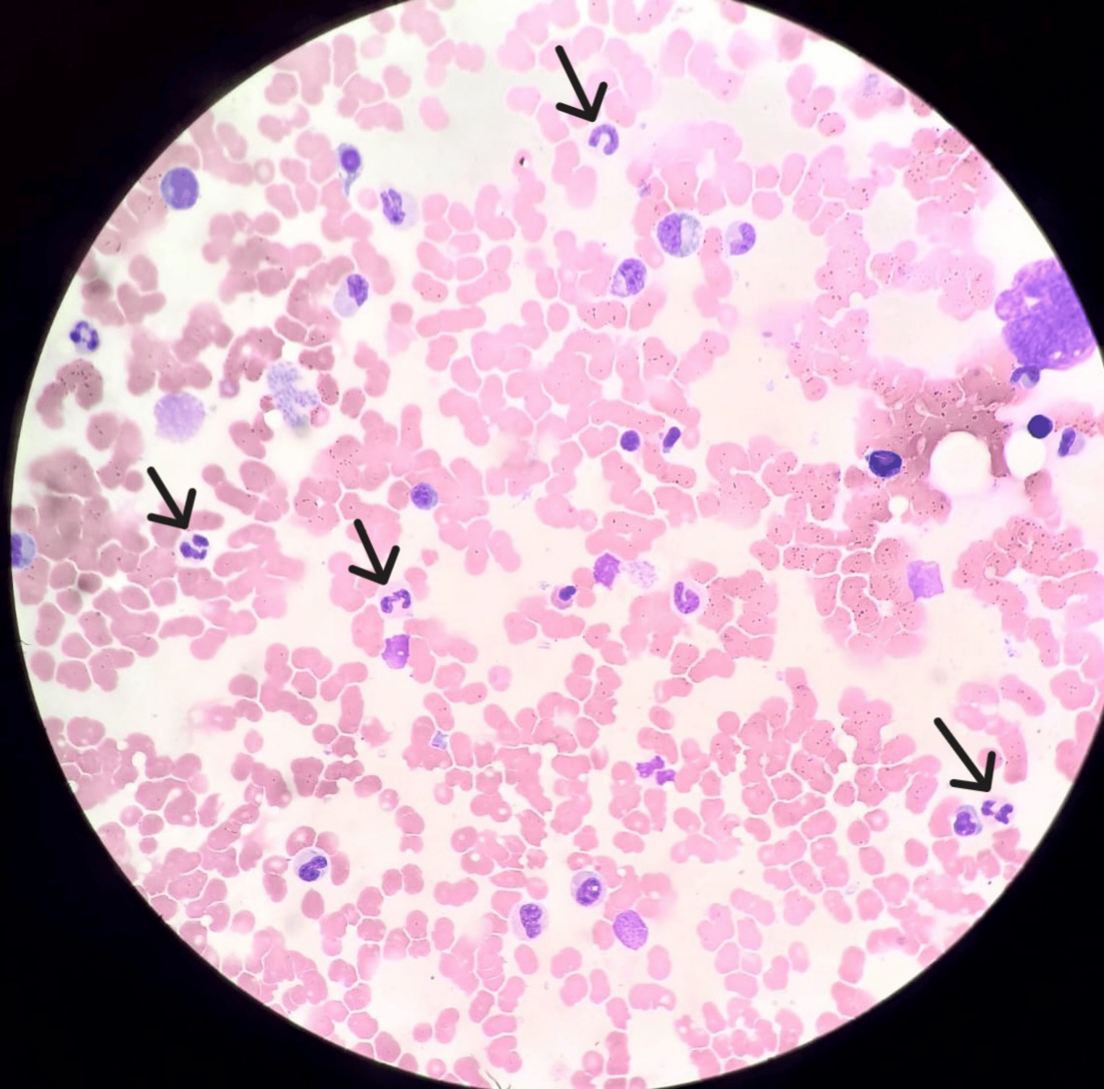
Dysgranulopoiesis - nuclear hyposegmentation Bone marrow smear under light microscopy, 60× oil immersion objective, using May-Grünwald-Giemsa staining Arrows indicate nuclear hyposegmentation in the neutrophils.

Granulocytic dysplasia was mainly characterized by cytoplasmic hypogranulation (36.62%), nuclear hyposegmentation (25.35%), and chromatin hypercondensation (14.08%). Multiple abnormalities were observed in 23 patients (32.39%) (Figure 3).

**Figure 4:**
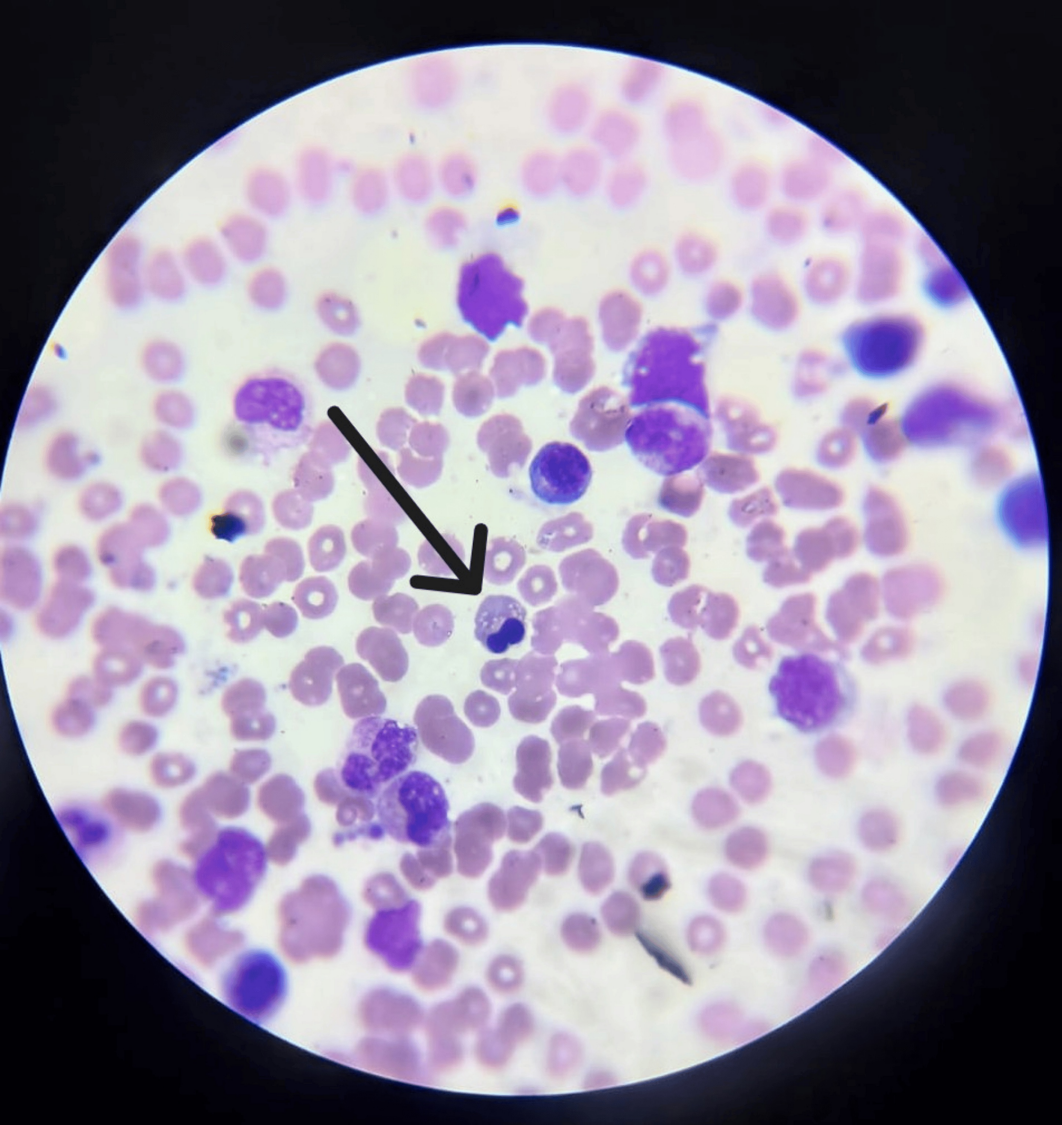
Erythroblastic dysplasia - budding nuclei, laminated cytoplasm Bone marrow smear under light microscopy, ×100 oil immersion objective, May-Grünwald-Giemsa staining The arrow indicates an acidophilic erythroblast with laminated cytoplasm and nuclear budding.

Dyserythropoiesis was predominantly characterized by erythroblasts with nuclear budding (39.44%), laminated cytoplasm (38.03%), and nucleo-cytoplasmic maturation asynchrony (19.72%) (Figure 4).

Based on the available clinical and laboratory data, dysmyelopoiesis was considered reactive in one-quarter of cases (25.35%). In contrast, the majority of cases were suggestive of an underlying malignant process (74.65%), particularly MDS. Multilineage dysplasia was observed in 31 patients. Blast counts were within normal limits in most cases; however, increased blast percentages were identified in a small number of patients, including one case consistent with acute leukemia (Table 4).

**Table 4:** Overall Conclusions of Bone Marrow Aspirate Examinations According to Probable Etiologies (71 cases)

Conclusion	Reactive etiology (n=18)	Malignant etiology (n=53)
Single lineage dysplasia		
Dysmegakaryopoiesis	0	4
Dysgranulopoiesis	8	8
Dyserythropoiesis	10	12
Multilineage dysplasia	0	31
Blast count		
No excess blasts (≤5%)	18	40
Type 1 blast excess (5–10%)	0	7
Type 2 blast excess (11–19%)	0	3
Acute leukemia (≥20%)	0	1
Chronic myelomonocytic leukemia	0	2

## Discussion

In the present study, the frequency of dysmyelopoiesis was 7.06%. This rate is lower than that reported in studies conducted in healthy individuals, where dysmyelopoiesis rates of up to 46% have been described when a 10% cutoff for dysplastic cells was applied [1,5]. Such differences may reflect variations in study populations, as well as differences in diagnostic criteria and interpretation.

The predominance of patients older than 60 years is consistent with the epidemiology of myelodysplastic syndromes, which mainly affect elderly individuals. However, the wide age range observed also highlights the heterogeneous nature of dysmyelopoiesis, which may occur in both malignant and reactive conditions [2,3].

Interpretation of dysmyelodysplastic features on bone marrow aspirates may vary between observers depending on the experience of the cytologist. This assessment is also influenced by pre-analytical and technical factors, including smear quality, staining procedures, and the type of microscope used [6]. These factors may contribute to inter-observer variability in the evaluation of dysplastic changes.

In the present study, smears were prepared immediately after aspiration to minimize morphology-related artifacts, in accordance with international recommendations stating that bone marrow samples intended for morphological assessment should be examined fresh and not remain exposed to anticoagulants for prolonged periods [7].

Cytopenias were a major finding in our series, with anemia being particularly frequent. The most frequent clinical presentation of MDS, formerly referred to as “refractory anemia,” is isolated non-regenerative anemia, most often macrocytic. This entity is primarily driven by clonal abnormalities of hematopoietic stem cells, commonly associated with recurrent somatic mutations, particularly in genes involved in RNA splicing. MDS are characterized by ineffective and dysplastic hematopoiesis, leading to peripheral cytopenias despite a typically normocellular or hypercellular bone marrow [3,8-10].

The mean hemoglobin level observed in this study was consistent with current literature findings. In MDS, anemia is a defining clinical feature, and hemoglobin thresholds used in prognostic scoring systems, such as the Revised International Prognostic Scoring System (IPSS-R), typically classify values <10 g/dL as clinically significant [8,9].

Dysplastic cells may be observed in either peripheral blood or bone marrow smears, exhibiting varying degrees of dysplasia. Blood smear examination may reveal macrocytosis associated with anisocytosis, a finding that is commonly reported in MDS [7,8,11], as observed in this study.

Erythroblastic hyperplasia was observed in 21.13% of cases (15/71), and in the case of non-regenerative anemia, it can be commonly associated with vitamin deficiencies, particularly folate (vitamin B9) and/or cobalamin (vitamin B12) deficiency. Overall, dyserythropoiesis was present in bone marrow examination in 73.23% of cases (52/71). Findings such as nuclear budding and maturation asynchrony are relatively frequent but nonspecific. In contrast, laminated cytoplasm in erythroblasts is a more characteristic feature of myelodysplastic syndromes, best appreciated at the polychromatophilic and orthochromatic stages, reflecting impaired hemoglobinization. This abnormality is often associated with a positive result for Perls’ stain (≥15% in the absence of SF3B1 mutation, ≥5% when present) [7,12]; however, this test was not performed in the present study due to technical limitations.

Dysgranulopoietic features can be identified in both peripheral blood and bone marrow smears. Abnormalities related to nuclear segmentation were frequent, observed in 48 patients (67.61%). These changes are often described as “Pelger-Huët-like” cells, characterized by neutrophils with bilobed or ring-shaped nuclei, and are commonly associated with chromatin hypercondensation, featuring dense clumps separated by paler areas [7]. Chromatin condensation is essential for genome stability and normally varies during the cell cycle, with further compaction during mitosis followed by decondensation in interphase; therefore, chromatin hypercondensation should not be present in interphase cells [13]. When observed outside physiological contexts, it may be associated with caryoschisis, characterized by small rod-like nuclear projections into the cytoplasm of neutrophils. Although this finding can be seen physiologically in females due to X-chromosome inactivation or after chemotherapy, its presence otherwise is suggestive of dysgranulopoiesis [3].

Neutrophil cytoplasmic granularity may be reduced (hypogranulation) or absent (degranulation). Conversely, abnormal coarse granulation can also be observed in neutrophils and/or eosinophils, including Chediak-Higashi-like granules, toxic granulation, and other atypical inclusions such as Döhle bodies. Although these abnormalities are classically associated with myelodysplastic syndromes, they may also be encountered in reactive conditions. Their presence is associated with an increased risk of infection, resulting from impaired bactericidal and bacteriostatic functions of myeloperoxidase, lactoferrin, and gelatinase, which are normally contained within cytoplasmic granules [14].

Dysmegakaryopoiesis can also be identified on peripheral blood smears. In this series, large platelets were observed in 14 patients, and circulating micromegakaryocytes in 3 patients. Megakaryocytic dysplasia is not the most frequently observed abnormality on bone marrow smears, but it holds significant diagnostic value. In this study, small-sized forms and hypolobated nuclei were the most common findings, observed in 26.76% and 11.27% of cases, respectively. Hypolobated megakaryocytes are characterized by a loss of the typical multilobulated nuclear configuration. This morphology is particularly suggestive of the 5q- syndrome, in which megakaryocytes typically exhibit small, round nuclei with abundant pale cytoplasm and are easily recognizable. In contrast, less marked hypolobation observed in other myelodysplastic syndromes is referred to as non-5q- morphology [15].

Peripheral blood typically reflects only the most mature circulating hematopoietic elements, whereas bone marrow evaluation captures the full spectrum of cellular maturation. Consequently, qualitative abnormalities are often more evident in bone marrow than in peripheral blood, where cytological heterogeneity is reduced, and immature or dysplastic populations may be underrepresented due to a physiological selection bias toward mature cells [16].

Bone marrow findings showed that most patients had either normal or increased cellularity. This apparent paradox, namely, the coexistence of peripheral cytopenias and preserved or increased marrow cellularity, is a well-known feature of ineffective hematopoiesis [17,18].

Reactive dysmyelopoiesis accounted for a non-negligible proportion of cases in this study, representing 18 of 71 cases (22.53%). In our setting, these cases were frequently associated with clinical contexts suggestive of reversible conditions, including vitamin deficiencies, severe infections, and post-chemotherapy status, and younger age, in which MDS are less commonly expected. Therapy-related hematopoietic dysplastic changes have been well described following cytotoxic treatments such as chemotherapy and radiotherapy, highlighting their non-clonal and potentially reversible nature [3].

Reactive dysplastic changes were also considered in patients without cytopenias or in those whose cytopenia profiles did not meet the diagnostic criteria for MDS. The abnormalities predominantly involved dyserythropoietic and dysgranulopoietic features. Nevertheless, distinguishing reactive from malignant dysmyelopoiesis remains challenging, particularly in the absence of cytogenetic or molecular investigations, as morphological dysplasia may lack specificity and can be observed in non-clonal conditions [18]. These patients, therefore, require close follow-up, especially regarding the evolution of cytopenias after correction or resolution of the suspected underlying condition.

Multilineage dysplasia predominated among cases suggestive of myelodysplastic syndromes in our series. This is consistent with the WHO classification, which defines MDS with multilineage dysplasia as a major disease subtype and a common morphological pattern reflecting involvement of multiple hematopoietic lineages [9].

The absence of excess blasts in most patients in our series suggests that suspected MDS cases were predominantly identified at relatively early stages of disease evolution. The occurrence of leukemic transformation was rare, with only one patient progressing to acute leukemia, highlighting the limited frequency of overt disease progression in our cohort. This observation is consistent with the known risk of leukemic transformation in MDS, which remains variable but is generally higher in advanced or high-risk subgroups, with reported rates of progression to acute myeloid leukemia of approximately 30-40% [19]. In this context, the WHO-based Prognostic Scoring System (WPSS) remains a useful tool for stratifying risk and estimating the likelihood of progression to acute myeloid leukemia in clinical practice, particularly when cytogenetic and molecular testing is unavailable. Unlike more recent models such as IPSS-R and IPSS-M (Molecular), WPSS relies primarily on morphological classification according to the WHO criteria, blast percentage, and transfusion dependency, all of which can be assessed using standard hematological and clinical data [20]. However, the absence of advanced diagnostic tools represents a major limitation, as these are essential for accurate classification and prognostic assessment according to current international standards.

## Conclusions

Dysmyelopoiesis remains a complex finding in routine hematological practice. In our series, dysplastic changes were identified in 7.06% of bone marrow examinations and were observed across a wide range of ages and clinical conditions. Although suggestive myelodysplastic syndromes represented a major cause of dysmyelopoiesis, a substantial proportion of cases were associated with non-clonal disorders, particularly suggestive nutritional deficiencies and infectious diseases. These findings highlight the heterogeneous nature of dysmyelopoiesis and demonstrate that dysplastic changes should not automatically be interpreted as evidence of a malignant hematological disorder. Accurate interpretation requires careful integration of morphological findings with clinical and laboratory data. In resource-limited settings, where cytogenetic and molecular investigations are often unavailable, this clinicopathological approach remains essential for appropriate diagnosis and patient management.

By providing original data from Madagascar, this study contributes to a better understanding of the spectrum of dysmyelopoiesis in sub-Saharan Africa and reinforces the need for a comprehensive diagnostic approach when evaluating dysplastic changes in bone marrow examinations.
